# Supplementary material for: Content-rich biological network constructed by mining PubMed abstracts
Source: BMC Bioinformatics. 2004 Oct 8;5:147. doi: 10.1186/1471-2105-5-147 (PMC528731; doi:10.1186/1471-2105-5-147)
Supplement: Additional File 2 — The original results of the above study (non-essential files are deleted to keep the file size under the limit set by BMC bioinformatics). [file 1471-2105-5-147-S2.bz2 › chilibotAdditionalFile2/dip05/653ID10446050E11044/html/PAPD_PAPK.html]

 


 **PAPD** and **PAPK** 
  
Found 6 abstracts in PubMed, retrieved 05.  
 

 What does Google say? 
 PDF only 
| .edu only 

---

**Interactive relationship** (e.g. stimulation, inhibition, etc)

**Neutral relationship**- **PapK** , an adaptor protein that joins the tip fibrillum to the pilus rod, formed only one complex with  **PapD**  DK.  Ref: 7909317 J Biol Chem, 1994

**Non-interactive relationship** (e.g. studied together, co-existance, homology, etc.)

- To gain additional insight into the interactive surfaces of HifA and HifB, we aligned HifA sequences from 20 different strains and then modelled the HifA structure based on the recently crystallized  **PapD**   **PapK**  complex.  Ref: 10760135 Mol Microbiol, 2000
- The structure of the  **PapD**   **PapK**  complex also suggests that during pilus biogenesis, every subunit completes the immunoglobulin like fold of its neighboring subunit via a mechanism termed donor strand exchange.  Ref: 10446050 Science, 1999
- The crystal structure of the  **PapD**   **PapK**  chaperone subunit complex, determined at 2.4 angstrom resolution, reveals that the chaperone functions by donating its G 1 beta strand to complete the immunoglobulin like fold of the subunit via a mechanism termed donor strand complementation.  Ref: 10446050 Science, 1999
- Thus, although Caf1M Caf1 subunit binding almost certainly involves the basic principle of donor strand complementation elucidated for the  **PapD**   **PapK**  complex, a key feature unique to the chaperones of this subfamily would appear to be capping via high affinity binding of an extended hydrophobic surface on the respective single subunits.  Ref: 11123684 Mol Microbiol, 2001
